# Planning peer assisted learning (PAL) activities in clinical schools

**DOI:** 10.1186/s12909-020-02289-w

**Published:** 2020-12-03

**Authors:** Annette Burgess, Christie van Diggele, Chris Roberts, Craig Mellis

**Affiliations:** 1 The University of Sydney, Faculty of Medicine and Health, Sydney Medical School - Education Office, Edward Ford Building A27, The University of Sydney, Sydney, NSW 2006 Australia; 2 The University of Sydney, Faculty of Medicine and Health, Sydney Health Professional Education Research Network, The University of Sydney, Sydney, Australia; 3 The University of Sydney, Faculty of Medicine and Health, The University of Sydney, Sydney, Australia; 4 The University of Sydney, Faculty of Medicine and Health, Sydney Medical School, Central Clinical School, Sydney, Australia

## Abstract

Peer Assisted Learning (PAL) is well accepted as an educational method within health professional education, involving a process of socialisation among students. PAL activities provide a framework whereby students are permitted to practice and develop their healthcare and teaching skills. However, the success of PAL activities is dependent upon two key factors: the *“agency”* of the individual students, that is, their willingness to participate; and importantly, the “*affordance”* of the activity, that is, the invitational quality provided by the clinical school. The purpose of this paper is to assist healthcare educators and administrators responsible for curriculum design, course co-ordination, and educational research, in developing their own PAL activities. Health professional students and junior health professionals leading or participating in PAL activities may also find the paper useful. Based on the authors’ collective experience, and relevant literature, we provide practical tips for the design, implementation and evaluation of PAL activities.

## Background

Peer Assisted Learning (PAL) activities encompass “*People from similar social groupings who are not professional teachers helping each other to learn and learning themselves by teaching”* [1]. Within health professional curricula, PAL is well accepted and utilised as an educational method, involving a process of socialisation, often with junior and senior students acting as tutees and tutors respectively. PAL activities provide a framework whereby students are permitted to practice and develop their healthcare and teaching skills [2, 3]. Through the contribution of students’ varied experiences, and the use of shared resources, students learn with and from each other. However, the success of PAL activities is dependent upon two key factors: the *“agency”* of the individual students (tutors and tutees), that is, their willingness to participate; and importantly, the “*affordance”* of the activity and the workplace, that is, the invitational quality provided by the clinical school [4]. To clarify terms used within this paper, we refer to the ‘tutor’ as the students who are assisting their student peers with their learning; and we refer to the ‘tutee’ as the students being assisted in their learning by the student peer ‘tutor’.

A common purpose for implementation of PAL programs is the requirement of students to teach in their future careers, and the provision of early opportunities in helping them to prepare for these roles [3, 5]. Initial knowledge and skills are gained through participation in tutor training programs, where students are taught how to teach. However, there is an additional responsibility for staff to ensure that appropriate opportunities are made available for health professional students to practice these teaching skills [3, 6]. The act itself of peer tutoring is thought to provide a rich learning opportunity for students to revise their own knowledge and skills [3, 7]. Additionally, PAL offers resource saving measures for universities and hospitals. Participation in PAL activities provides additional support in preparation for assessments, and may address gaps in curriculum delivery [8].

The purpose of this paper is to assist healthcare educators and administrators responsible for curriculum design, course co-ordination, and education research, in the development of their own PAL activities. Health professional students and junior health professionals leading or participating in PAL activities may also find the paper useful. Based on the authors’ collective experience, and relevant literature, we aim to provide tips for the design, implementation and evaluation of PAL activities.

### Design of PAL activities

Engagement in tasks, and a commitment to the PAL activities from both students and faculty is increased through careful planning and advanced preparation [1, 3, 9]. The following areas should be considered:

#### How does the PAL activity align with the curriculum?

It is important to consider how the PAL activity aligns with the curriculum, and how it might be embedded within the curriculum in future years. PAL programs afford opportunities not otherwise available within traditional healthcare curricula. Learning involves a process of preparation before class; and socialisation during class, supported by structured teaching methods and communication tools, where students’ development of knowledge and skills are jointly constructed. Health professional students have many differing roles across their clinical school, university, and beyond [7]. PAL activities that are employed should make explicit the professional expectations of students as healthcare graduates, highlighting the alignment with the current curriculum (for example, preparation for clinical assessment), and requisite graduate competencies [10].

#### Who will lead the PAL activity?

Planning and implementation of PAL activities requires detailed preparation and support, usually by a team of academic and administrative staff members. Although PAL activities require a team approach in terms of organisation and implementation, it is necessary to have a ‘lead’ for each activity, with an outline of responsibilities. Generally, the role of the lead, in consultation with key stakeholders entails co-ordination of: design, planning, curriculum alignment, delivery, and evaluation. The lead needs to maintain the direction of the PAL activity, ensuring good communication between team members. Although there are reports of ‘student led’ PAL activities, our own experience suggests that the support of staff and the university/hospital helps to formalise and promote the program, and gain ‘buy-in’ from students [4].

#### What are the resource costs?

Well run PAL activities require high levels of administrative time. The training of staff and students, creation of learning resources, and evaluation design, all require faculty time [3, 9, 11]. The expertise, time commitment for administration of the PAL activities, and associated costs, including timetabling, room bookings, notifications, catering, distribution and collection of evaluation forms, and trouble shooting, should not be underestimated. Most PAL activities require additional financial support, particularly when PAL is not embedded within the curriculum. Preparation of a budget for each PAL activity will assist in justification of resource investment by the School.

### Student participation in PAL activities

#### Who tutors who?

Tutor and tutee roles with PAL activities are not necessarily fixed, and at different times, the tutor may also be a tutee [3, 9, 11]. For example, a reciprocal form of PAL occurs within the same cohort of students, providing a ‘same year dyadic PAL’ [1, 12, 13]. There can also be variations in the dynamics of the PAL activities. For example:

- Direct peer-to-peer (students tutoring or assessing within the same cohort)
- Near peer (senior students tutoring or assessing junior students)

#### Recruitment of participants?

Student participation in PAL activities can exist on a voluntary or compulsory basis. Ten Cate and Durning posit that PAL participation should be “*part of the regular mandatory programme”* as a means to increase efficiency [12]. Our own experience suggests that this is certainly true for *some* PAL activities, particularly when the participating student cohort is preparing for summative examinations. However, some PAL activities are better as a combination of both mandatory and voluntary participation. For example, mandatory participation for student tutees, but voluntary participation for student tutors who are enthusiastic.

#### What motivates students to participate in PAL activities?

Tutor motivation for joining PAL activities can include both intrinsic and extrinsic rewards [3]. While some universities have reported using monetary rewards for participation in PAL programs, *intrinsic rewards* feature predominantly in the literature [3]. These include: altruistic reasons for helping other students; gaining insights and understanding in assessment processes; developing a greater understanding of the topic; and the development of teaching and assessment skills [3].

#### Can your PAL activity be interprofessional?

Currently, there are limited examples of interprofessional activities within university healthcare education, with most being discipline specific [14]. Although there is often a preference for activities within individual disciplines, there is a need to consider how interprofessional education can be enhanced to mirror, and better prepare students for the complex healthcare systems in which they will work [15]. Early training and experience in interprofessional activities has many potential benefits, including improvements in leadership, collaboration and communication between healthcare teams, ultimately improving patient safety [16–18]. Although challenges include logistics, resource allocation, and differing terminology, there are many associated benefits [19]. Importantly, an interprofessional context provides a dynamic tool to increase participants’ understanding of the various roles of healthcare professionals, and shape opportunities for interprofessional activities [19, 20]. The provision of networking opportunities helps to build relationships between healthcare disciplines, and positively impacts the culture of organisations. For example, small group interprofessional activities, where participants are able to share their experiences with students from other healthcare professions, has the potential to improve communication skills, and provide a deeper understanding of the multidisciplinary work required in patient care.

### Training and preparation for the PAL activities

The skill of teaching is best acquired through a sequence of training, practice and feedback [5]. However, recent systematic reviews of health professional student teacher training programs reveal common deficits: inadequate assessment of participants prior to participation as peer tutors; lack of practice opportunities; and lack of meaningful feedback to facilitate improvement in teaching skills [3, 20–22]. Evidence suggests that those who are provided with adequate training prior to teaching find the task more enjoyable, and feel better prepared for future teaching roles [23, 24].

#### What type of training is useful?

It is important to consider the tutor’s prior training and experience. For example, tutor roles may include: creation of learning resources, small group teaching to consolidate prior knowledge and skills, formative assessment, and provision of feedback [3, 25]. A needs analysis can help to inform the design of a training package. Training may include pre-reading/preparation, a formal training session, and an assessment of competence in terms of content knowledge and/or teaching ability [3, 19, 26].

#### Training models and the use of frameworks

Training commonly focuses on the basic principles of teaching, including theory with opportunities for practice. Often tutor training is quite short, and focused on the specific PAL activity, with more general tutor training being offered over an extended period of time. Areas of difficulty to address during tutor training include: clarification of the tutor/assessor role; marking criteria; and the discomfort around tutoring and provision of feedback to peers. Students value the use of frameworks that may assist them in teaching and examining their peers, for example, Peyton’s four step approach to teaching a skill [27], or ISBAR [28] in communicating a clinical handover.

Another useful framework is Pendlenton’s model of providing feedback [29]. The use of feedback, and the teaching of skills in giving and receiving feedback, is essential to most training programs. Although giving and receiving feedback is an essential component of a lifelong career within the health professions, it is a skill seldom taught at university, and reported as deficient in the healthcare workforce [19–22, 30, 31]. Feedback promotes self-reflection, and reinforces the positive aspects of an individual’s performance.

### Implementing PAL activities

PAL activities within clinical schools provide students with a “safe” environment, enabling them to take on and practice teaching and formative assessment roles. Participation in PAL activities develops new “professional” skills (educational expertise), while at the same time, consolidating students’ medical knowledge and skills. These learning activities assist in the development of students’ emerging identities as future health professionals, with teaching responsibilities. There are numerous PAL activities described in recent healthcare education literature [3, 5, 7]. Drawing on our own experience in design and implementation of PAL activities that align with the curriculum, we illustrate four examples in Table 1. Over time, these PAL activities have become embedded in the curriculum, and broadened for implementation across faculties, with some implemented within an interprofessional context. Our PAL activities include:

- **PAL Formative clinical long case examinations**
  The formative clinical long case examination requires students to undertake an unobserved history taking and physical examination of a patient for one hour, after which students are assessed for 20 min by two co-examiners: one faculty member, and one student. The ‘examinee’ then leaves the room for 10 min while the co-examiners discuss the feedback that is verbally provided, and led by the student co-examiner. These formative examinations are designed to inform students of their progress and specific areas of need for improvement, in preparation for their high stakes barrier examination. It is mandatory for all students to participate, both as co-examiners, and as examinees. A 30 min training/information session is provided to students. The formative long case PAL activity and evaluation has been previously reported [32–35].
- **PAL Formative Objective Structured Clinical Examination (OSCE)**
  In the formative OSCE, students are assessed at five stations, including communication, examination, and procedural skills stations. Senior students (Year 4 students, who are in their final year) act as assessors of their peers; senior students (Year 3) act as simulated patients; and junior students (Years 1 and 2) are formatively assessed. It is mandatory for all junior students to participate, while senior students participate on a voluntary basis [36, 37].
  The PAL formative OSCE activity has been previously described [36, 37].
- **PAL Peer tutoring: a student led program**
  The Peer tutoring program is a formal, student-led program, supported by administrative staff (e.g. emailing, room booking), and endorsed by faculty. Senior students (Years 3 and 4) tutor junior students (Years 1 and 2). Participation is voluntary for all students. Four tutees are assigned to a pair of tutors. Year 3 students tutor Year 1 students, and Year 4 students tutor Year 2 students. Tutorials are one hour in duration, covering clinically relevant content that is aligned with the current teaching block. They are designed to supplement existing teaching to enhance tutees’ knowledge base. Delivery of the tutorials is structured and interactive, with a 20 min briefing, 20 min ward teaching, and 20 min debriefing. The PAL peer tutoring program and evaluation has been previously reported [4].
- **PAL Peer Teacher Training program**
  The Peer Teacher Training (PTT) program is designed to support health professional students in the development of their teaching, assessment and feedback skills, preparation for PAL activities, and future healthcare professional practice. Delivered as a six module, blended learning program, participants are provided with theoretical background, and opportunities for active participation in small group interprofessional learning teams. Participation in the PTT program is voluntary, and interprofessional. Allied health, nursing, pharmacy, medicine and dentistry students are invited to participate. Various designs of the PTT program to suit both students and junior health professionals, including the Clinical Teacher Training program, have been previously reported [19, 22, 38].

**Table 1:** Examples of PAL activities within The University of Sydney Medical School

“Tutor”/“Examiner” PARTICIPANTS	Tutee” /“Examinee” PARTICIPANTS	PARTICIPATION VOLUNTARY/COMPULSORY	RESOURCE REQUIREMENTS	TRAINING PROVIDED	EVALUATION
**FORMATIVE CLINICAL LONG CASE EXAMINATIONS**					
**Description:** Senior students (Year 3 and Year 4) act as co-examiners assessors of their direct peers, alongside a faculty member. Student examiners are responsible for finding patients on wards [32–35].					
**Purpose**: Designed to prepare students for their summative long case examinations by informing them of their strengths and weaknesses					
Year 3 and Year 4 medical students.	Year 3 and Year 4 medical students.	Participation is compulsory for all Year 3 and Year 4 students.	∙ Hospital ‘in-patients’ ∙ Faculty ∙ Students ∙ Small rooms ∙ Exam administrator ∙ One senior faculty member is required to act as a co-examiner at each set of long case examinations	Students (examiners and examinees) are provided with a 30 min briefing.	Students value the experience as examiners and examinees. Acting as a co-examiner provides insights into the exam process. Student co-examiners are more lenient markers than faculty. Student co-examiners find it difficult to provide honest and critical feedback
**FORMATIVE OSCE EXAMINATIONS**					
**Description**: Students are examined at five OSCE stations to assess communication, physical examination or procedural skills. Year 4 students assess Year 1 and 2 students. Year 3 students act as simulated patients [36, 37].					
**Purpose:** Designed to prepare Year 1 and Year 2 students for their summative OSCEs, in terms of the OSCE process, and to inform them of specific areas of knowledge and skills that need strengthening.					
Year 3: simulated patients Year 4: assessors	Year 1 and Year 2 medical students	Participation is compulsory for all Year 1 and Year 2 students; and voluntary for Year 3 and Year 4 students.	∙ Small rooms ∙ Exam administrator ∙ Preparation of OSCE material, including OSCE questions, marking sheets. ∙ Faculty review and facilitation of feedback	Student assessors (Year 4) and simulated patients (Year 3) are provided with a 30 min briefing. Year 1 and 2 students are provided with written information.	Year 3 Student simulated patients believed the exercise improved their knowledge base, confidence in clinical skills, and developed their understanding of the patient-doctor relationship. They found it helpful in preparing for their own future examinations. It reduced the logistical demands and cost to clinical schools with limited resources. Year 4 examiners found peer assessment to be a very useful learning activity. Although students felt confident in the accuracy of their marking, they consistently rated their peers as performing better than do faculty. However, students need further training in how to globally assess a fellow student’s overall performance objectively to provide accurate feedback.
**PEER TUTORING (A STUDENT LED PROGRAM)**					
**Description:** Senior students (Years 3 and 4) tutor junior students (Years 1 and 2). Four tutees are assigned to a pair of tutors. Year 3 students tutor Year 1 students, and Year 4 students tutor Year 2 students. Tutorials are one hour long, covering clinically relevant content that is aligned with the current teaching block. Tutorials are designed to supplement existing teaching to enhance tutee’s knowledge base. Tutorial delivery is interactive, with 20 min briefing, 20 min ward teaching, and 20 min debriefing [4].					
**Purpose:** The Peer tutoring program is a formal, student led program, supported by staff, designed to provide additional support to junior students, and allow senior students the opportunity to practice teaching skills, and reinforce their own knowledge.					
Year 3 and Year 4 students as tutors (Year 3 students tutor Year 1 students; and Year 4 tutor Year 2 students)	Year 1 and Year 2 medical students	Participation is voluntary for all students	∙ Students ∙ Small rooms ∙ Faculty review of tutor material	Student tutors and tutees are provided with a one hour briefing by the senior student leads.	The peer tutoring program provided a framework within the medical curriculum for senior students to practice and improve their medical knowledge and teaching skills. Concurrently, junior students were provided with a valuable learning experience that they reported as being qualitatively different to traditional teaching by faculty.
**PEER TEACHER TRAINING (PTT) PROGRAM (interprofessional)**					
**Description:** Delivered as a six module, interprofessional, blended learning program, participants are provided with theoretical background, and opportunities for active participation in small group interprofessional learning teams [19, 22, 38].					
**Purpose:** The Peer Teacher Training (PTT) program is designed to support health professional students in the development of their teaching, assessment and feedback skills, in preparation for PAL activities, and future health professional practice.					
Senior students from all healthcare (medicine, nursing, pharmacy, health sciences, dentistry) faculties are invited to attend	Senior students practice teaching and clinical handover in small groups with other senior students	Participation is voluntary for all students	∙ Administrative support ∙ Faculty & alumni (‘graduates’ of previous PTT programs) to facilitate small groups	Students are required to complete pre-reading and preparation before attending the face-to-face class	The flipped learning, interprofessional format was successful in developing students’ skills, competence and confidence in teaching, assessment, communication and feedback. Importantly, participation increased students’ awareness and understanding of the various roles of health professionals.

### Evaluation of PAL activities

The evaluation of PAL activities should constructively align with the project aims. For example, if the principle aim of the PAL activity relates to increasing student knowledge, then this should be included in the evaluation [39]. If an aim is to develop student teaching ability, this should be assessed with the evaluation. PAL outcomes are usually multifaceted and require mixed methods in the evaluation. Evaluation protocols may require submission to the university or hospital ethics committee. Thoughtful evaluation design, considered at the early planning stage, allows the PAL co-ordinator to:

- demonstrate the worth of the program
- justify expenditure
- direct improvements
- apply for grants to widen participation
- demonstrate improvements to student outcomes

#### Types of evaluation

As indicated in systematic literature reviews, most literature reflects participant perception of PAL activities [3, 5, 7, 20, 21]. Although this is usually positive, it may not be reflective of all benefits or challenges of PAL activities. Topping has suggested that the “monitoring and control” of new PAL initiatives, should not be underestimated [1]. Methods of evaluation that may be less subjective than questionnaires and focus groups include: observation by staff, researchers, peers or simulated patients; pre-and post-tests; student knowledge retention; and comparisons in marking ability of faculty and students [3].

## Conclusion

This paper reflected on the design, implementation, and evaluation requirements of PAL activities within university clinical schools. PAL activities offer a dynamic tool in helping to shape the social constructs of learning. Participation in PAL activities help students to recognise and develop their future roles and responsibilities as health professionals with teaching and assessment responsibilities. Although sometimes resource intensive, once established and standardised, PAL activities offer many benefits to the students (both tutors and tutees), faculty, and institutions.

### Take-home message

**Table:** 

• Provision of tutor training programs is essential for student peer tutors. Students value the use of frameworks to assist them in tutoring. For example, Pendleton’s model of providing feedback. • Staff should ensure that appropriate practical opportunities are made available for students to apply and practice their new tutoring skills. • PAL activities require careful planning, design, implementation and evaluation. • Review of existing literature is useful when considering the design of PAL activities within your clinical school. • Provision of an interprofessional context for PAL activities increases participants’ understanding of the various roles of health professionals, provides networking opportunities, and provides a deeper understanding of the multi-disciplinary work required in patient care. • Evaluation should provide evidence of the benefits to students, the worth of the program, and needs for improvement.	

## Data Availability

Not applicable.

## Abbreviations

PTT: Peer Teacher Training
PAL: Peer Assisted Learning
OSCE: Objective Structured Clinical Examination
